# Supplementary material for: Chromatin Landscape Dynamics in the Early Development of the Plant Parasitic Nematode Meloidogyne incognita
Source: Front Cell Dev Biol. 2021 Dec 6;9:765690. doi: 10.3389/fcell.2021.765690 (PMC8685519; doi:10.3389/fcell.2021.765690)
Supplement: Supplementary file 1 [file DataSheet1.PDF]

Chromatin landscape dynamics in development of the plant parasitic nematode *Meloidogyne incognita*

Supplementary Figure 1 | Two-step antibody validation for ChIP-Seq. Examples of two-step antibody validation adapted from (Cosseau *et al.*, 2009): Western blot detection of **(A)** acetylated H3 at lysine 27 (H3K27ac), **(B)** monomethylated H4 at lysine 20 (H4K20me1) and **(C)** trimethylated H3 at lysine 27 (H3K27me3). qPCR validation on immunoprecipitated chromatin from *M. incognita*, with various volumes (0-16 µL) of **(D)** anti-H3K27 acetyl and **(E)** anti-H4K20 monomethyl antibodies. The percent input recovery (%IR) was calculated from the targeted amount of DNA and normalized with respect to the percent input recovery for the housekeeping gene. **(A)** and **(D)** show examples of successful validation for both western blotting and ChIP-titration, whereas **(B)** and **(E)** were validated only on western blotting, and **(C)** was not validated at the first step.

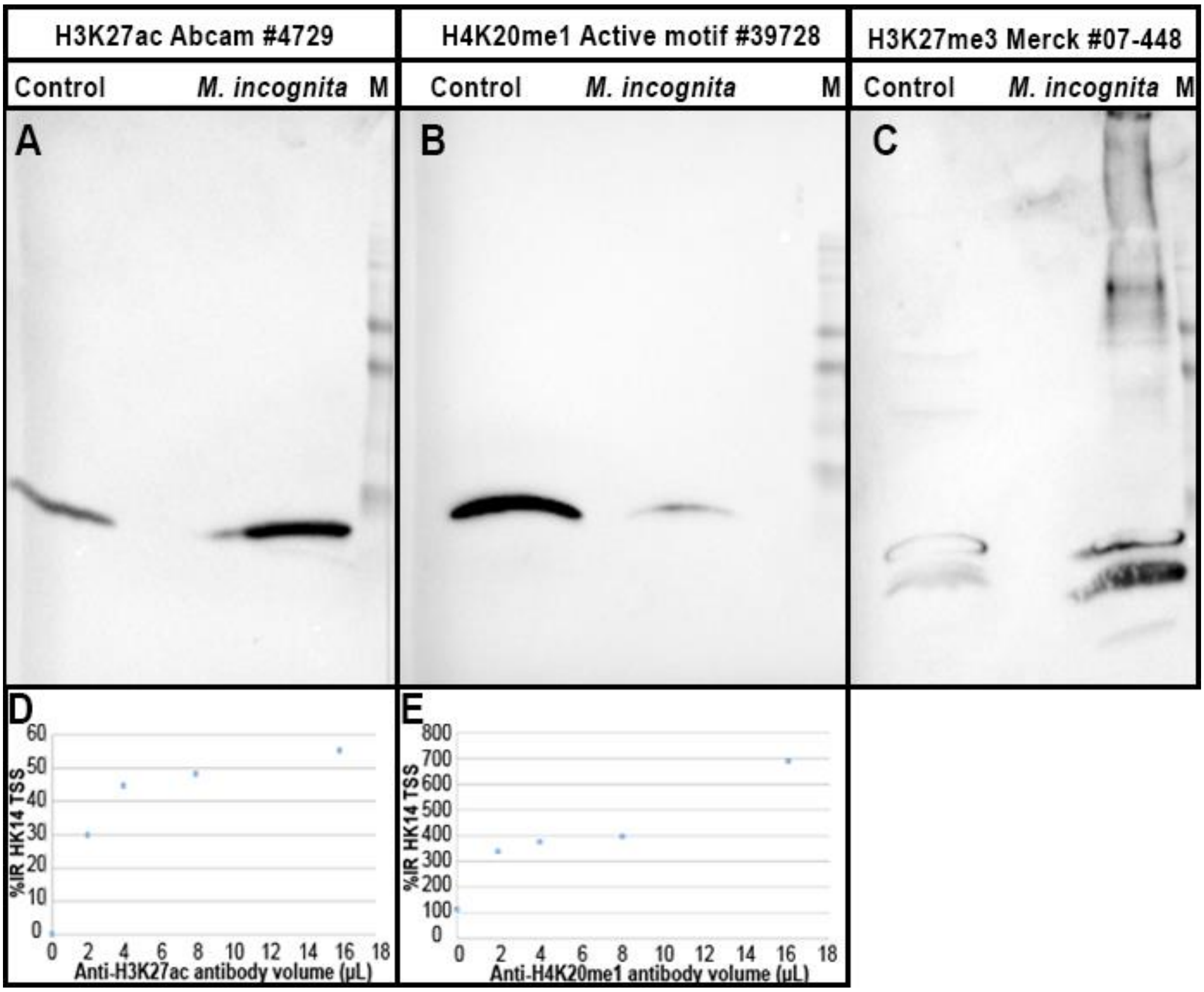

Supplementary Figure 2 | H3K4me3, H3K9me3, H3K27ac, H27me3 and H4K20me1 histone modifications on the *M. incognita* genome. Triplicate tracks of histone modifications are illustrated on *M. incognita* at high resolution. Sequence reads for **(A)** H3K4me3 (blue, scaffold Minc3s00004), **(B)** H3K9me3 (red, scaffold Minc3s00013), **(C)** H3K27ac (pink, scaffold Minc3s00038), **(D)** H3K27me3 (green, scaffold Minc3s00003) and **(E)** H4K20me1 (black, scaffold Minc3s00007) samples were visualized in IGV software. Values shown on the y axis represent the relative enrichment of ChIP-Seq signals obtained with PeakRanger (peaks correspond to read counts after background/input subtraction). For each histone modification, the three biological replicates (rep1, rep2 and rep3) are shown. Each track contains information from one biological replicate of eggs.

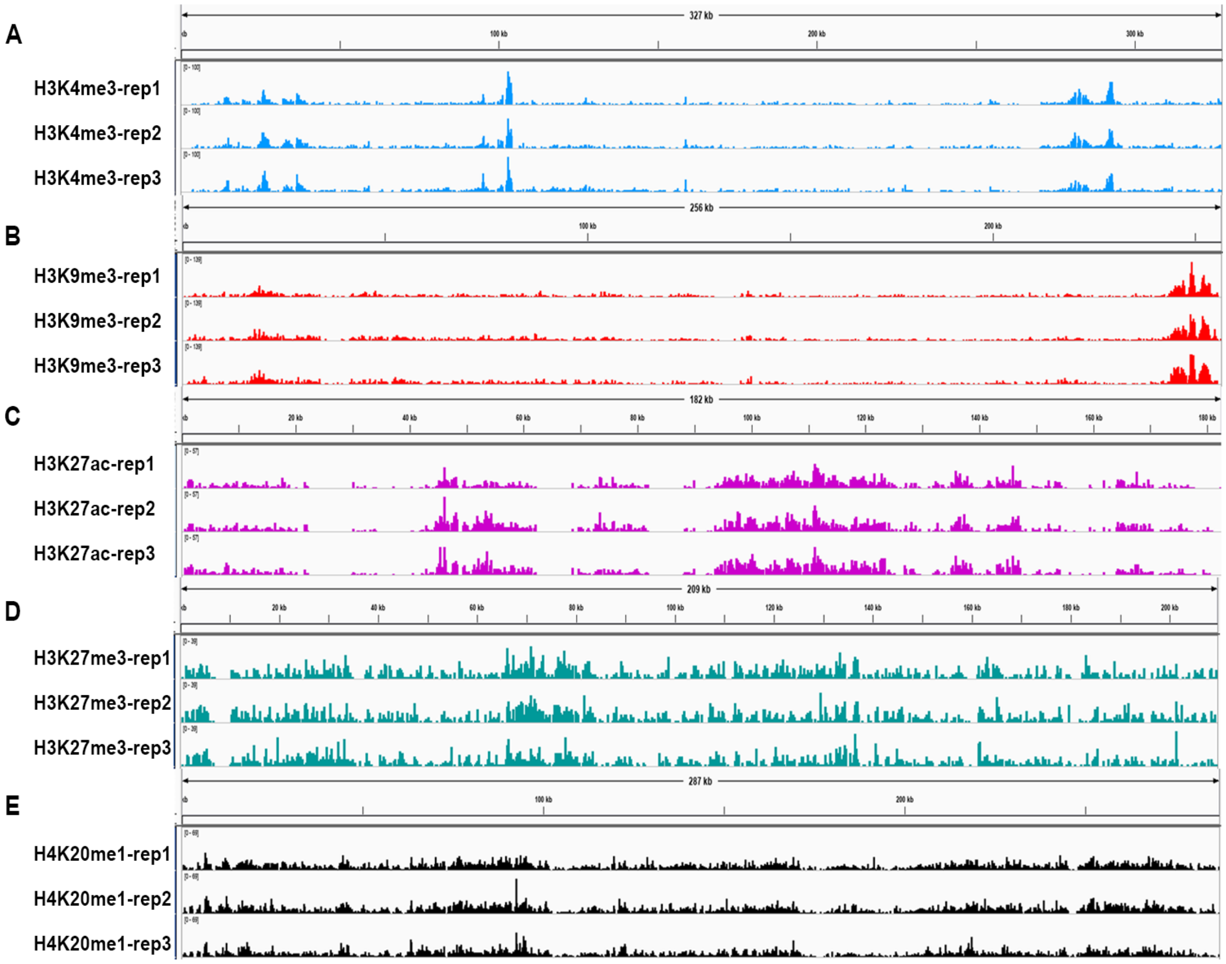

Supplementary Figure 3 | Distinct epigenetic landscapes at the transposable element (TE) and protein coding-gene annotations in *M. incognita*. **(A)** Illustration of H3K9me3 enrichment in association with TE. Screenshot of the full scaffold Minc3s00875 with selected tracks for H3K9me3 (red), TE and gene annotations (dark blue). **(B)** Illustration of H3K4me3 enrichment in association with expressed protein-coding genes. Screenshot of the full scaffold Minc3s03894 with selected tracks for H3K4me3 (sky-blue), gene annotations (dark blue) and eggs transcripts (RNA-seq; grey). Samples were visualized in IGV software. Values shown on the y axis represent the relative enrichment of ChIP-Seq signals obtained with PeakRanger (peaks corresponding to read counts, normalized by the percent input method). Each track contains information from one biological replicate of eggs.

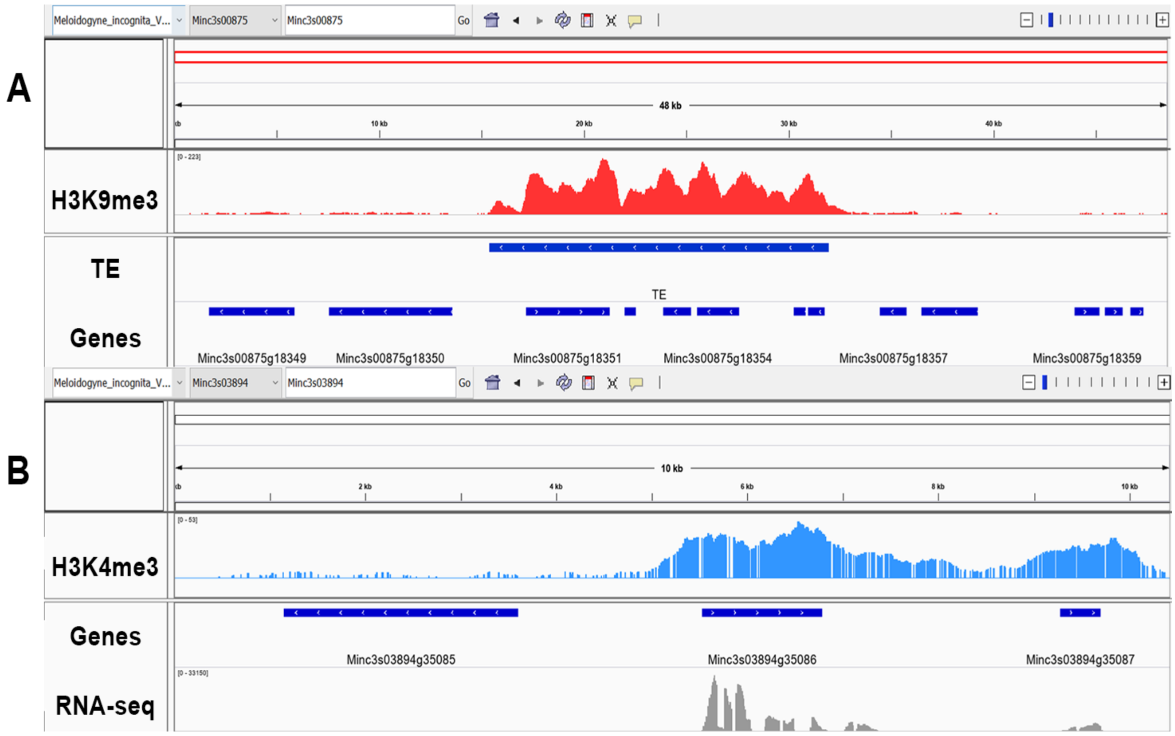

Supplementary Table 1 | Antibodies selected and tested for ChIP-seq analysis on *M. incognita*. Antibodies were selected based on their availability, their validation in *C. elegans* and their validation as ChIP-grade when possible. Each antibody was tested by a two-step validation process, as described by Cosseau et al., 2009. The amount of antibody used was determined by titration at the saturation point.

Epitope	Supplier	Catalog #	Batch #	Clonality	Western Blot	ChIP-qPCR	Antibody amount
H4K20monomethyl	Active Motif	39728	#21115004	Monoclonal	Yes	No	Not validated
H4K20trimethyl	Active Motif	39672	#25815004	Monoclonal	Yes	No	Not validated
H3K9acetyl	Active Motif	61664	#04415001	Monoclonal	Yes	No	Not validated
H3K56acetyl	Active Motif	61062	#09811001	Monoclonal	Yes	No	Not validated
H3K4dimethyl	Abcam	ab32356	#GR84714-4	Monoclonal	Yes	No	Not validated
H4K20trimethyl	Abcam	ab9053	#GR86824-1	Polyclonal	Yes	No	Not validated
H3K27acetyl	Abcam	ab4729	#GR150367-2	Polyclonal	Yes	Yes	4µL
H3K27trimethyl	Epigentek	A-4039	#503019	Polyclonal	Yes	Yes	4µL
H3K4trimethyl	Merck Millipore	04-745	#2392291 and #2452485	Monoclonal	Yes	Yes	8µL
H3K9trimethyl	Abcam	ab8898	#GR306402-2	Polyclonal	Yes	Yes	8µL
H4K20monomethyl	Abcam	ab9051	#GR158874-1	Polyclonal	Yes	Yes	8µL
H3K36trimethyl	Abcam	ab9050	#GR300387-1	Polyclonal	No	No	Not validated
H3K27monomethyl	Merck Millipore	07-448	#JBC1361682	Polyclonal	No	No	Not validated
H3K27acetyl	Millipore	07-360	#Q2209321	Polyclonal	No	No	Not validated
H3K27acetyl	Active Motif	39134	#1613007	Polyclonal	No	No	Not validated
H3K9acetyl	Merck Millipore	07-352	#3089126	Polyclonal	No	No	Not validated

Supplementary Table 2 | Genome wide distribution and heatmap enrichment values of histone modifications in relation to annotations for the *M. incognita* genome. The distribution of histone modifications was analyzed with ChromstaR, which calculated the spatial enrichment in histone modifications for different available genomic annotations. As for **Figure 1** and **Figure 3**, enrichment heatmaps are 5x10 matrix representing 5 histone modifications (H3K4me3, H3K9me3, H3K27ac, H3K27me3 and H4K20me1) and 10 genomic annotated elements (CDS, exon, five prime UTR, gene, mRNA, ncRNA, rRNA, TE, three prime UTR and tRNA). The value is significant when different from 0, on a scale ranging from -2.5 (depletion) to +2.5 (enrichment) for genomic annotations or on a scale ranging from -4 (depletion) to +4 (enrichment) for TE subfamily annotations. Three biological replicates have been treated jointly to identify common histone modification enrichment for *M. incognita* eggs or J2s.

**Genomic annotations-Eggs**										
	**CDS**	**Exon**	**five_prime_UTR**	**gene**	**mRNA**	**ncRNA**	**rRNA**	**TE**	**three_prime_UTR**	**tRNA**
**[H3K27ac]**	0.593847	0.544234	1.206274614	0.085474	0.085628	0.271903	-2.37745	-1.05412	1.118039783	1.230605
**[H3K27me3]**	0.084787	0.021477	0.535782258	-0.36076	-0.36364	-2.03489	-2.37745	-0.36933	0.300766277	1.577795
**[H3K4me3]**	1.030751	0.996232	2.3774463	0.49535	0.497193	1.004841	-2.29156	-0.89106	1.598413609	0.594057
**[H3K9me3]**	0.557259	0.483529	0.40948824	-0.02978	-0.02953	0.118898	-1.29719	1.1925	0.55814676	0.76425
**[H4K20me1]**	0.73769	0.672601	0.810275305	0.250439	0.249232	0.647357	-0.92288	-0.31648	1.005354863	1.583244
**Genomic annotations-J2s**										
	**CDS**	**Exon**	**five_prime_UTR**	**gene**	**mRNA**	**ncRNA**	**rRNA**	**TE**	**three_prime_UTR**	**tRNA**
**[H3K27ac]**	0.549953	0.507382	1.244931815	0.057163	0.05725	-0.50421	-2.37745	-1.03104	1.131059334	1.316713
**[H3K27me3]**	0.063236	0.001765	0.507573723	-0.38143	-0.38492	-0.87308	-1.8837	-0.25775	0.312793121	1.668387
**[H3K4me3]**	1.037922	1.001521	2.360837758	0.501616	0.50356	0.959814	-2.33658	-0.85961	1.587233378	0.583515
**[H3K9me3]**	0.461032	0.388188	0.367597032	-0.10437	-0.10595	0.505835	-0.84126	0.945317	0.485297665	1.179359
**[H4K20me1]**	0.733562	0.666895	0.792943432	0.238715	0.238788	0.543093	-0.87299	-0.08039	0.968456143	1.178395
**TE subfamily annotations-Eggs**										
	**Helitron**	**LARD**	**LINE**	**LTR**	**Maverick**	**MITE**	**SINE**	**TIR**	**TRIM**	
**[H3K27ac]**	-3.97013	-3.97013	-2.249617005	-3.15757	-3.97013	-0.65125	-3.97013	-0.64773	-1.840409558	
**[H3K27me3]**	-3.97013	-2.04298	-1.442895607	-1.62133	-3.97013	0.051955	-3.97013	-0.0386	-1.151471195	
**[H3K4me3]**	-3.97013	-0.88356	-2.886165839	-1.27284	-2.5512	-0.18155	-0.43996	-1.41349	-0.39751685	
**[H3K9me3]**	1.835407	1.31478	1.428431498	1.836058	2.011493	0.411148	-3.97013	0.521018	1.202987972	
**[H4K20me1]**	0.395977	-3.97013	-0.888879879	-1.73229	-2.62766	0.063488	-3.97013	-0.04589	0.213474749	
**TE subfamily annotations-J2s**										
	**Helitron**	**LARD**	**LINE**	**LTR**	**Maverick**	**MITE**	**SINE**	**TIR**	**TRIM**	
**[H3K27ac]**	-3.97013	-3.97013	-2.80258946	-2.1701	-3.97013	-0.68652	-3.97013	-0.62819	-1.007087651	
**[H3K27me3]**	-3.97013	-1.16886	-0.974236871	-1.32788	-3.68379	0.145148	-3.97013	0.055645	-0.970494531	
**[H3K4me3]**	-3.97013	-0.72792	-2.238046163	-1.30898	-2.41391	-0.14859	-0.48499	-1.39993	-0.362501647	
**[H3K9me3]**	2.100282	0.90321	0.971078619	1.733719	1.888238	-0.08096	-3.97013	-0.1247	0.659739912	
**[H4K20me1]**	-0.87589	-3.97013	0.03647596	-0.73333	-0.42179	0.159098	-3.97013	0.029554	-0.247463773	

Supplementary Table 3 | *M. incognita* Egg-overexpressed genes orthologs in *C. elegans* . GO enrichment analysis showed specific terms associated with orthologous egg-overexpressed genes. Protein family/subfamily and classes were obtained using PANTHER and detailed for each *M. incognita* and *C. elegans* gene, with their GO ID.

Gene_ID	GO_ID	Cele_ortholog	PANTHER_Family_Subfamily	PANTHER_Protein_Class
Minc3s00022g01429	GO:0042302	-	-	-
Minc3s00024g01555	GO:0005515	-	-	-
Minc3s00059g03033	GO:0008270	-	-	-
Minc3s00122g05254	GO:0005515	-	-	-
Minc3s00175g06764	GO:0006470,GO:0004725	-	-	-
Minc3s00228g08060	GO:0005515	-	-	-
Minc3s00235g08214	GO:0005921	-	-	-
Minc3s00270g09053	GO:0006334,GO:0005634,GO:0000786,GO:0003677,GO:0046982	-	-	-
Minc3s00496g13313	GO:0042302	-	-	-
Minc3s00617g15162	GO:0006508,GO:0008237,GO:0008270,GO:0004222	-	-	-
Minc3s00743g16779	GO:0006468,GO:0004672,GO:0005524,GO:0004674,GO:0004713,GO:0016772	-	-	-
Minc3s00778g17229	GO:0005921	-	-	-
Minc3s00778g17230	GO:0005921	-	-	-
Minc3s00948g19200	GO:0046872	-	-	-
Minc3s01033g20041	GO:0005921	-	-	-
Minc3s01509g24378	GO:0003677	-	-	-
Minc3s02210g28873	GO:0003677	-	-	-
Minc3s02531g30493	GO:0006334,GO:0000786,GO:0003677,GO:0046982	-	-	-
Minc3s03074g32677	GO:0005488	-	-	-
Minc3s03635g34388	GO:0005921	-	-	-

Minc3s04120g35526	GO:0006468,GO:0004672,GO:0005524,GO:0004674,GO:0004713,GO:0016772	-	-	-
Minc3s04120g35527	GO:0005515,GO:0008270	-	-	-
Minc3s06804g40340	GO:0005515	-	-	-
Minc3s07231g40874	GO:0006334,GO:0000786,GO:0003677,GO:0046982	-	-	-
Minc3s09509g43272	GO:0006334,GO:0005634,GO:0000786,GO:0003677,GO:0046982	-	-	-
Minc3s11337g44818	GO:0006355,GO:0003700,GO:0043565,GO:0003677	-	-	-
Minc3s00781g17277	GO:0005524,GO:0008026,GO:0003676,GO:0004386	A0FLQ6	DNA POLYMERASE THETA (PTHR10133:SF41)	DNA-directed DNA polymerase(PC00018)
Minc3s01717g25828	GO:0006260,GO:0006139,GO:0000166,GO:0003677,GO:0003887,GO:0003676	C7IVS4	DNA POLYMERASE ALPHA CATALYTIC SUBUNIT (PTHR45861:SF1)	DNA metabolism protein(PC00009)
Minc3s00307g09837	GO:0000910,GO:0000226,GO:0008017	G5ED58	FASCETTO-RELATED (PTHR19321:SF0)	non-motor microtubule binding protein(PC00166)
Minc3s00398g11618	GO:0007017,GO:0006184,GO:0051258,GO:0005874,GO:0043234,GO:0005525,GO:0003924,GO:0005200	G5EDD4,H2L2E8,O18154,P34690,P52274,P91873,P91910,Q19490,Q20221	TUBULIN ALPHA CHAIN (PTHR11588:SF182)	tubulin(PC00228)

Minc3s00491g13212	GO:0007017,GO:0006184,GO:0051258,GO:0005874,GO:0043234,GO:0005525,GO:0003924,GO:0005200	G5EDD4,H2L2E8,O18154,P34690,P52274,P91873,P91910,Q19490,Q20221	TUBULIN ALPHA CHAIN (PTHR11588:SF182)	tubulin(PC00228)
Minc3s01886g26965	GO:0007017,GO:0006184,GO:0051258,GO:0005874,GO:0043234,GO:0005525,GO:0003924,GO:0005200	G5EDD4,H2L2E8,O18154,P34690,P52274,P91873,P91910,Q19490,Q20221	TUBULIN ALPHA CHAIN (PTHR11588:SF182)	tubulin(PC00228)
Minc3s00077g03777	GO:0005524,GO:0003677	G5EEW5,P91494	TRANSCRIPTION TERMINATION FACTOR 2 (PTHR45626:SF22)	-
Minc3s00645g15561	GO:0007018,GO:0005871,GO:0003777,GO:0005524,GO:0008017	G5EGS3	KINESIN MOTOR DOMAIN-CONTAINING PROTEIN (PTHR24115:SF929)	microtubule binding motor protein(PC00156)
Minc3s01717g25830	GO:0006468,GO:0004672,GO:0005524,GO:0004674,GO:0004713,GO:0016772	O01427,G5EDL3	NON-SPECIFIC SERINE/THREONINE PROTEIN KINASE (PTHR24350:SF0)	non-receptor serine/threonine protein kinase(PC00167)
Minc3s00093g04328	GO:0006275,GO:0043626,GO:0003677,GO:0030337	O02115	PROLIFERATING CELL NUCLEAR ANTIGEN (PTHR11352:SF0)	DNA polymerase processivity factor(PC00015)
Minc3s00026g01674	GO:0006260,GO:0006270,GO:0005634,GO:0042555,GO:0005524,GO:0003677,GO:0003678	O16297	DNA REPLICATION LICENSING FACTOR MCM7 (PTHR11630:SF26)	DNA metabolism protein(PC00009)
Minc3s00001g00047	GO:0007017,GO:0006184,GO:0051258,GO:0005874,GO:0043234,GO:0005525,GO:0003924,GO:0005200	O17921,P12456,P41937,P52275,Q18817	TUBULIN BETA CHAIN-RELATED (PTHR11588:SF357)	tubulin(PC00228)

Minc3s00035g02063	GO:0007017,GO:0006184,GO:0051258,GO:0005874,GO:0043234,GO:0005525,GO:0003924,GO:0005200	O17921,P12456,P41937,P52275,Q18817	TUBULIN BETA CHAIN-RELATED (PTHR11588:SF357)	tubulin(PC00228)
Minc3s00761g16988	GO:0007017,GO:0006184,GO:0051258,GO:0005874,GO:0043234,GO:0005525,GO:0003924,GO:0005200	O17921,P12456,P41937,P52275,Q18817	TUBULIN BETA CHAIN-RELATED (PTHR11588:SF357)	tubulin(PC00228)
Minc3s00990g19633	GO:0007017,GO:0006184,GO:0051258,GO:0005874,GO:0043234,GO:0005525,GO:0003924,GO:0005200	O17921,P12456,P41937,P52275,Q18817	TUBULIN BETA CHAIN-RELATED (PTHR11588:SF357)	tubulin(PC00228)
Minc3s01465g24052	GO:0006468,GO:0005524,GO:0004674,GO:0016772,GO:0004672	O18209	MEMBRANE-ASSOCIATED TYROSINE- AND THREONINE-SPECIFIC CDC2-INHIBITORY KINASE (PTHR11042:SF99)	non-receptor serine/threonine protein kinase(PC00167)
Minc3s00887g18516	GO:0006836,GO:0016021,GO:0005328	O44752	SODIUM: NEUROTRANSMITTER SYMPORTER FAMILY (PTHR11616:SF295)	primary active transporter(PC00068)
Minc3s00914g18817	GO:0006334,GO:0005634,GO:0000786,GO:0003677,GO:0046982	P04255,Q27484,Q27876,Q27894	HISTONE H2B-RELATED (PTHR23428:SF70)	histone(PC00118)
Minc3s02531g30496	GO:0006334,GO:0005634,GO:0000786,GO:0003677,GO:0046982	P04255,Q27484,Q27876,Q27894	HISTONE H2B-RELATED (PTHR23428:SF70)	histone(PC00118)
Minc3s00628g15312	GO:0006334,GO:0005634,GO:0000786,GO:0003677,GO:0046982	P09588,Q27485	HISTONE H2A (PTHR23430:SF342)	histone(PC00118)
Minc3s00156g06294	GO:0006468,GO:0005515,GO:0004672,GO:0005524,GO:0004674,GO:0004713,GO:0016772	P34331,Q9N2L7,Q20845	SERINE/THREONINE-PROTEIN KINASE PLK-1 (PTHR24345:SF52)	non-receptor serine/threonine protein kinase(PC00167)

Minc3s01168g21362	GO:0006468,GO:0005515,GO:0004672,GO:0005524,GO:0004674,GO:0004713,GO:0016772	P34331,Q9N2L7,Q20845	SERINE/THREONINE-PROTEIN KINASE PLK-1 (PTHR24345:SF52)	non-receptor serine/threonine protein kinase(PC00167)
Minc3s02806g31633	GO:0006468,GO:0005515,GO:0004672,GO:0005524,GO:0004674,GO:0004713,GO:0016772	P34331,Q9N2L7,Q20845	SERINE/THREONINE-PROTEIN KINASE PLK-1 (PTHR24345:SF52)	non-receptor serine/threonine protein kinase(PC00167)
Minc3s01318g22720	GO:0006468,GO:0004672,GO:0005524,GO:0004674,GO:0004713,GO:0016772	P34556	CYCLIN-DEPENDENT KINASE 1 (PTHR24056:SF334)	non-receptor serine/threonine protein kinase(PC00167)
Minc3s00047g02557	GO:0055114,GO:0009186,GO:0016491,GO:0046914	P42170	RIBONUCLEOSIDE-DIPHOSPHATE REDUCTASE SUBUNIT M2 (PTHR23409:SF18)	reductase(PC00198)
Minc3s00668g15869	GO:0055114,GO:0009186,GO:0016491,GO:0046914	P42170	RIBONUCLEOSIDE-DIPHOSPHATE REDUCTASE SUBUNIT M2 (PTHR23409:SF18)	reductase(PC00198)
Minc3s00014g00879	GO:0006457,GO:0003755	P52013,P52014	PPIASE CYCLOPHILIN-TYPE DOMAIN-CONTAINING PROTEIN-RELATED (PTHR11071:SF513)	chaperone(PC00072)
Minc3s06127g39373	GO:0006414,GO:0005737,GO:0005525,GO:0003924,GO:0003746	P53013	ELONGATION FACTOR 1-ALPHA 2 (PTHR23115:SF170)	translation factor(PC00223)
Minc3s00142g05891	GO:0006334,GO:0005634,GO:0000786,GO:0003677,GO:0046982	P62784	HISTONE H4 (PTHR10484:SF162)	histone(PC00118)
Minc3s00270g09051	GO:0006334,GO:0005634,GO:0000786,GO:0003677,GO:0046982	P62784	HISTONE H4 (PTHR10484:SF162)	histone(PC00118)
Minc3s00463g12755	GO:0006334,GO:0005634,GO:0000786,GO:0003677,GO:0046982	P62784	HISTONE H4 (PTHR10484:SF162)	histone(PC00118)

Minc3s00914g18818	GO:0006334,GO:0005634,GO:0000786,GO:0003677,GO:0046982	P62784	HISTONE H4 (PTHR10484:SF162)	histone(PC00118)
Minc3s02861g31856	GO:0006334,GO:0005634,GO:0000786,GO:0003677,GO:0046982	P62784	HISTONE H4 (PTHR10484:SF162)	histone(PC00118)
Minc3s01484g24182	GO:0005515	Q09397	F-BOX DOMAIN-CONTAINING PROTEIN (PTHR16134:SF123)	-
Minc3s02004g27710	GO:0005515	Q09397	F-BOX DOMAIN-CONTAINING PROTEIN (PTHR16134:SF123)	-
Minc3s01706g25758	GO:0003729	Q09599	HISTONE RNA HAIRPIN-BINDING PROTEIN (PTHR17408:SF0)	RNA metabolism protein(PC00031)
Minc3s00407g11796	GO:0005515	Q09967,Q21629	LDL RECEPTOR REPEAT-CONTAINING PROTEIN EGG-1-RELATED (PTHR24270:SF39)	apolipoprotein(PC00052)
Minc3s04557g36534	GO:0010389,GO:0000079,GO:0051726,GO:0005634,GO:0019901	Q10654	G2/MITOTIC-SPECIFIC CYCLIN-B3 (PTHR10177:SF214)	kinase activator(PC00138)
Minc3s01016g19878	GO:0006508,GO:0004190	Q17569	AT13091P (PTHR12917:SF1)	aspartic protease(PC00053)
Minc3s00393g11542	GO:0009103,GO:0008830	Q17993	DTDP-4-DEHYDRORHAMNOSE 3,5-EPIMERASE (PTHR21047:SF2)	epimerase/racemase(PC00096)
Minc3s01358g23096	GO:0006457,GO:0006950,GO:0005524,GO:0051082	Q18688,Q22235	HEAT SHOCK PROTEIN 83 (PTHR11528:SF34)	Hsp90 family chaperone(PC00028)
Minc3s04772g36976	GO:0006457,GO:0006950,GO:0005524,GO:0051082	Q18688,Q22235	HEAT SHOCK PROTEIN 83 (PTHR11528:SF34)	Hsp90 family chaperone(PC00028)
Minc3s00578g14541	GO:0055114,GO:0016491	Q19000	LP01339P (PTHR10696:SF50)	hydroxylase(PC00122)
Minc3s07100g40720	GO:0006334,GO:0005634	Q19007	LD21576P (PTHR11875:SF7)	chromatin/chromatin-binding, or -regulatory protein(PC00077)
Minc3s00718g16490	GO:0005921	Q19746	INNEXIN-3 (PTHR11893:SF20)	gap junction(PC00105)

Minc3s00388g11452	GO:0007018,GO:0035556,GO:0005871,GO:0003777,GO:0005524,GO:0008017,GO:0005515,GO:0005543	Q21025,Q9XVH4,G5ECF4	PROTEIN CBG04771 (PTHR22988:SF39)	non-receptor serine/threonine protein kinase(PC00167)
Minc3s00703g16321	GO:0007018,GO:0005871,GO:0003777,GO:0005524,GO:0008017	Q21025,Q9XVH4,G5ECF4	PROTEIN CBG04771 (PTHR22988:SF39)	non-receptor serine/threonine protein kinase(PC00167)
Minc3s00082g03930	GO:0006260,GO:0006270,GO:0005634,GO:0042555,GO:0005524,GO:0003677,GO:0003678	Q21902	DNA REPLICATION LICENSING FACTOR MCM5 (PTHR11630:SF42)	DNA metabolism protein(PC00009)
Minc3s00070g03518	GO:0007275,GO:0016020,GO:0004872	Q27874	INTEGRIN BETA-PS (PTHR10082:SF60)	integrin(PC00126)
Minc3s03732g34636	GO:0007275,GO:0016020,GO:0004872	Q27874	INTEGRIN BETA-PS (PTHR10082:SF60)	integrin(PC00126)
Minc3s01870g26869	GO:0006281,GO:0006259,GO:0005524,GO:0008094,GO:0003677,GO:0000166,GO:0017111,GO:0003684	Q95Q25	DNA REPAIR PROTEIN RAD51 HOMOLOG 1 (PTHR22942:SF39)	DNA metabolism protein(PC00009)
Minc3s10902g44454	GO:0006281,GO:0006259,GO:0005524,GO:0008094,GO:0003677,GO:0000166,GO:0003684	Q95Q25	DNA REPAIR PROTEIN RAD51 HOMOLOG 1 (PTHR22942:SF39)	DNA metabolism protein(PC00009)
Minc3s01096g20678	GO:0006457	Q95Q60	PEPTIDYLPROLYL ISOMERASE (PTHR46046:SF6)	-
Minc3s04628g36696	GO:0007076,GO:0005488	Q95Y84	CONDENSIN-2 COMPLEX SUBUNIT D3 (PTHR14222:SF1)	chromatin/chromatin-binding, or -regulatory protein(PC00077)

Minc3s00273g09139	GO:0007155,GO:0007156,GO:0016020,GO:0005886,GO:0005509,GO:0005515	Q967F4	CADHERIN-89D (PTHR24026:SF109)	cadherin(PC00057)
Minc3s00156g06297	GO:0007094	Q9NGT3	MITOTIC SPINDLE ASSEMBLY CHECKPOINT PROTEIN MAD2A (PTHR11842:SF11)	-
Minc3s01168g21364	GO:0007094	Q9NGT3	MITOTIC SPINDLE ASSEMBLY CHECKPOINT PROTEIN MAD2A (PTHR11842:SF11)	-
Minc3s06470g39859	GO:0005634,GO:0018024,GO:0005515,GO:0008270	Q9NH52	HISTONE-LYSINE N-METHYLTRANSFERASE CG1716-RELATED (PTHR22884:SF413)	-
Minc3s00273g09132	GO:0007049,GO:0031105,GO:0005525	Q9U334	SEPTIN-7 (PTHR18884:SF126)	cytoskeletal protein(PC00085)
Minc3s01304g22615	GO:0000166,GO:0005515,GO:0005543,GO:0003676	Q9XTT4	ANILLIN-LIKE PROTEIN 1 (PTHR21538:SF11)	non-motor actin binding protein(PC00165)
Minc3s02959g32218	GO:0006260,GO:0006270,GO:0005634,GO:0042555,GO:0005524,GO:0003677,GO:0003678,GO:0000166,GO:0017111	Q9XVR7	DNA REPLICATION LICENSING FACTOR MCM3 (PTHR11630:SF46)	DNA metabolism protein(PC00009)
Minc3s00583g14633	GO:0006260,GO:0006270,GO:0005634,GO:0042555,GO:0005524,GO:0003677,GO:0003678	Q9XXI9	DNA REPLICATION LICENSING FACTOR MCM2 (PTHR11630:SF44)	DNA metabolism protein(PC00009)
Minc3s04131g35557	GO:0006270,GO:0005634,GO:0042555,GO:0003678,GO:0005524,GO:0003677	Q9XXI9	DNA REPLICATION LICENSING FACTOR MCM2 (PTHR11630:SF44)	DNA metabolism protein(PC00009)

Supplementary Table 4 | *M. incognita* J2-overexpressed genes orthologs in *C. elegans* . GO enrichment analysis showed specific terms associated with orthologous J2-overexpressed genes. Protein family/subfamily and classes were obtained using PANTHER and detailed for each *M. incognita* and *C. elegans* gene, with their GO ID.

Gene_ID	GO_ID	Cele_ortholog	PANTHER_Family_Subfamily	PANTHER_Protein_Class
Minc3s00019g01229	GO:0006355,GO:0003700	-	-	-
Minc3s00043g02384	GO:0035023,GO:0005089,GO:0005515	-	-	-
Minc3s00061g03127	GO:0008270,GO:0005515	-	-	-
Minc3s00069g03459	GO:0006355,GO:0007179,GO:0005622,GO:0005667,GO:0005515,GO:0003700	-	-	-
Minc3s00077g03765	GO:0005509	-	-	-
Minc3s00088g04180	GO:0003676,GO:0046872	-	-	-
Minc3s00096g04404	GO:0055085,GO:0016021	-	-	-
Minc3s00097g04473	GO:0071805,GO:0016020,GO:0005267	-	-	-
Minc3s00130g05555	GO:0006355,GO:0003700	-	-	-
Minc3s00140g05827	GO:0007186,GO:0016021,GO:0004930	-	-	-
Minc3s00164g06502	GO:0006355,GO:0005634,GO:0043565,GO:0003677,GO:0003700,GO:0008270	-	-	-
Minc3s00221g07904	GO:0006355,GO:0043401,GO:0005634,GO:0003707,GO:0003700	-	-	-
Minc3s00288g09480	GO:0016021	-	-	-
Minc3s00326g10264	GO:0005515	-	-	-
Minc3s00354g10848	GO:0006355,GO:0007179,GO:0005622,GO:0005667,GO:0005515,GO:0003700	-	-	-
Minc3s00398g11622	GO:0005634,GO:0043565	-	-	-
Minc3s00457g12628	GO:0008270,GO:0005515	-	-	-

Minc3s00491g13226	GO:0005515	-	-	-
Minc3s00492g13244	GO:0006979,GO:0055114,GO:0004602	-	-	-
Minc3s00510g13525	GO:0008270,GO:0003676	-	-	-
Minc3s00570g14437	GO:2001070,GO:0030246	-	-	-
Minc3s00662g15772	GO:0055085,GO:0016021	-	-	-
Minc3s00692g16166	GO:0005576,GO:0005184	-	-	-
Minc3s00716g16468	GO:0006836,GO:0016021,GO:0005328	-	-	-
Minc3s00840g17971	GO:0006508,GO:0031012,GO:0008237,GO:0008270,GO:0004222	-	-	-
Minc3s00939g19096	GO:0055114,GO:0009055,GO:0016705,GO:0020037,GO:0005506	-	-	-
Minc3s00956g19273	GO:0007165,GO:0000159,GO:0008601,GO:0005488	-	-	-
Minc3s00956g19274	GO:0007165,GO:0000159,GO:0008601,GO:0005488	-	-	-
Minc3s00972g19447	GO:0006355,GO:0005634,GO:0043565,GO:0003700	-	-	-
Minc3s01022g19938	GO:0003676,GO:0046872	-	-	-
Minc3s01046g20158	GO:0008270,GO:0003676	-	-	-
Minc3s01201g21658	GO:0007186,GO:0016021,GO:0004930,GO:0004984	-	-	-
Minc3s01225g21869	GO:0008152,GO:0016491	-	-	-
Minc3s01351g23033	GO:0006355,GO:0005634,GO:0043565,GO:0003700	-	-	-
Minc3s01366g23188	GO:0008152,GO:0016491	-	-	-
Minc3s01372g23238	GO:0006457	-	-	-
Minc3s01381g23323	GO:0005615	-	-	-

Minc3s01565g24761	GO:0006355,GO:0005634,GO:0008270,GO:0003700,GO:0043565	-	-	-
Minc3s01914g27167	GO:0006810,GO:0016020,GO:0005215	-	-	-
Minc3s02059g28024	GO:0006812,GO:0016021,GO:0019829,GO:0000166,GO:0046872	-	-	-
Minc3s02207g28857	GO:0046488,GO:0016307	-	-	-
Minc3s02336g29530	GO:0009058,GO:0030170,GO:0003824	-	-	-
Minc3s02421g29936	GO:0005576,GO:0030570	-	-	-
Minc3s02528g30466	GO:0006355,GO:0043565,GO:0003700	-	-	-
Minc3s02949g32176	GO:0055114,II-requiring] activity	-	-	-
Minc3s03179g33059	GO:0008270,GO:0005515	-	-	-
Minc3s03481g33968	GO:0006508,GO:0004190	-	-	-
Minc3s03981g35228	GO:0006836,GO:0016021,GO:0005328	-	-	-
Minc3s05010g37427	GO:0006836,GO:0016021,GO:0005328	-	-	-
Minc3s05633g38563	GO:0006979,GO:0055114,GO:0004602	-	-	-
Minc3s05791g38836	GO:0015671,GO:0019825,GO:0020037,GO:0005506	-	-	-
Minc3s06204g39490	GO:0015671,GO:0019825,GO:0020037,GO:0005506	-	-	-
Minc3s06694g40195	GO:0006835,GO:0016020,GO:0017153	-	-	-
Minc3s07432g41118	GO:0006508,GO:0008270,GO:0004181	-	-	-

Minc3s08224g42031	GO:0006810,GO:0016020,GO:0005215	-	-	-
Minc3s08788g42599	GO:0006470,GO:0016311,GO:0016791,GO:0008138,GO:0004725	-	-	-
Minc3s10768g44352	GO:0008270	-	-	-
Minc3s11959g45263	GO:0000166,GO:0003676	-	-	-
Minc3s04056g35365	GO:0008270	A0A061AD47	LIM ZINC-BINDING DOMAIN-CONTAINING PROTEIN (PTHR24206:SF95)	actin or actin-binding cytoskeletal protein(PC00041)
Minc3s05702g38667	GO:0007186,GO:0016021,GO:0004930	D0FY26	G_PROTEIN_RECEP_F1_2 DOMAIN-CONTAINING PROTEIN (PTHR47632:SF4)	transmembrane signal receptor(PC00197)
Minc3s00388g11447	GO:0007186,GO:0007166,GO:0016021,GO:0016020,GO:0004930,GO:0004888	G5EBF7	SECRETIN/CLASS B GPCR (PTHR45620:SF37)	transmembrane signal receptor(PC00197)
Minc3s00858g18163	GO:0006811,GO:0055085,GO:0006813,GO:0051260,GO:0016020,GO:0008076,GO:0005216,GO:0005515,GO:0005249	G5EC00,G5EEK7,G5EFC3	BTB DOMAIN-CONTAINING PROTEIN (PTHR11537:SF252)	voltage-gated ion channel(PC00241)
Minc3s00400g11657	GO:0016787	G5EC18	SERINE/THREONINE-PROTEIN PHOSPHATASE (PTHR11668:SF485)	protein phosphatase(PC00195)

Minc3s01228g21896	GO:0055114,GO:0009055,GO:0016705,GO:0020037,GO:0005506	G5ECD0,H2KY69,O02627,O02628,O16220,O44649,O44650,O44651,O44652,O44655,O44656,O44657,O44658,O45364,O61204,O61935,O62377,O62378,Q9N5I1,G5ED86,Q27505,Q27506,Q27507,Q27531,G5EE22,O02641,O16362,O16482,O16670,O16671,O16672,O16673,O44704,O44706,O45659,Q27470,Q27471,Q27482,Q27499,Q965T7,Q9N4Q4,H2KYS3,P90771	CYTOCHROME P450 FAMILY (PTHR24300:SF114)	oxygenase(PC00177)

Minc3s03068g32645	GO:0055114,GO:0009055,GO:0016705,GO:0020037,GO:0005506	G5ECD0,H2KY69,O02627,O02628,O16220,O44649,O44650,O44651,O44652,O44655,O44656,O44657,O44658,O45364,O61204,O61935,O62377,O62378,Q9N5I1,G5ED86,Q27505,Q27506,Q27507,Q27531,G5EE22,O02641,O16362,O16482,O16670,O16671,O16672,O16673,O44704,O44706,O45659,Q27470,Q27471,Q27482,Q27499,Q965T7,Q9N4Q4,H2KYS3,P90771	CYTOCHROME P450 FAMILY (PTHR24300:SF114)	oxygenase(PC00177)
Minc3s01543g24582	GO:0006508,GO:0004252	G5ECN9	NEUROENDOCRINE CONVERTASE 2 (PTHR42884:SF13)	serine protease(PC00203)
Minc3s01914g27161	GO:0055085,GO:0016021	G5EDA7	BETA-ALANINE TRANSPORTER (PTHR24064:SF494)	secondary carrier transporter(PC00258)
Minc3s00088g04176	GO:0005515	G5EDE9	RELA-ASSOCIATED INHIBITOR (PTHR24164:SF4)	-
Minc3s01581g24883	GO:0005515	G5EDE9	RELA-ASSOCIATED INHIBITOR (PTHR24164:SF4)	-
Minc3s00844g18027	GO:0055114,GO:0008152,GO:0016491	G5EDW8,G5EDM5	HYDROXYCARBOXYLATE DEHYDROGENASE B-RELATED (PTHR11091:SF0)	oxidoreductase(PC00176)
Minc3s03783g34797	GO:0055114,GO:0008152,GO:0016491	G5EDW8,G5EDM5	HYDROXYCARBOXYLATE DEHYDROGENASE B-RELATED (PTHR11091:SF0)	oxidoreductase(PC00176)

Minc3s00785g17335	GO:0006810,GO:0016020,GO:0005415	G5EE77,H2KYD6	SODIUM/NUCLEOSIDE COTRANSPORTER (PTHR10590:SF20)	transporter(PC00227)
Minc3s01126g20962	GO:0006810,GO:0016020,GO:0005415	G5EE77,H2KYD6	SODIUM/NUCLEOSIDE COTRANSPORTER (PTHR10590:SF20)	transporter(PC00227)
Minc3s00582g14616	GO:0032313,GO:0005515,GO:0005543,GO:0005097	G5EEB5	TBC (TRE-2/BUB2/CDC16) DOMAIN FAMILY (PTHR22957:SF289)	GTPase-activating protein(PC00257)
Minc3s00125g05325	GO:0000166,GO:0003676	G5EFS2	RNA-BINDING PROTEIN MUSASHI HOMOLOG RBP6 (PTHR48032:SF6)	-
Minc3s01795g26361	GO:0000166	G5EFS2	RNA-BINDING PROTEIN MUSASHI HOMOLOG RBP6 (PTHR48032:SF6)	-
Minc3s03777g34773	GO:0000166,GO:0003676	G5EFS2	RNA-BINDING PROTEIN MUSASHI HOMOLOG RBP6 (PTHR48032:SF6)	-
Minc3s01007g19793	GO:0007186,GO:0016021,GO:0005887,GO:0004930,GO:0004993	G5EGH0	DOPAMINE RECEPTOR 2-RELATED (PTHR24247:SF241)	G-protein coupled receptor(PC00021)
Minc3s00007g00478	GO:0005515	H2KYZ5	UBIQUITIN-LIKE DOMAIN-CONTAINING PROTEIN (PTHR15140:SF6)	chaperone(PC00072)
Minc3s00358g10928	GO:0003333,GO:0016020,GO:0015171	H2KZG9	AMINO ACID TRANSPORTER (PTHR11785:SF302)	transporter(PC00227)
Minc3s00097g04463	GO:0006355,GO:0046983	H2KZZ2	BHLH DOMAIN-CONTAINING PROTEIN (PTHR45776:SF2)	-
Minc3s01899g27048	GO:0007186,GO:0016021,GO:0004965,GO:0004930	H2L0Q3	G_PROTEIN_RECEP_F3_4 DOMAIN-CONTAINING PROTEIN (PTHR10519:SF42)	G-protein coupled receptor(PC00021)
Minc3s04545g36507	GO:0007186,GO:0016021,GO:0004965,GO:0004930	H2L0Q3	G_PROTEIN_RECEP_F3_4 DOMAIN-CONTAINING PROTEIN (PTHR10519:SF42)	G-protein coupled receptor(PC00021)
Minc3s00107g04769	GO:0006094,GO:0004611,GO:0017076,GO:0005525,GTP activity	O02286,O44906	PHOSPHOENOLPYRUVATE CARBOXYKINASE (GTP)-RELATED (PTHR11561:SF0)	kinase(PC00137)

Minc3s01581g24877	GO:0006508,GO:0008234	O16288,O16289,P43508,P43509,O45466,P25807,P43507,P43510	PEPT_C1 DOMAIN-CONTAINING PROTEIN (PTHR12411:SF281)	cysteine protease(PC00081)
Minc3s02882g31948	GO:0006508,GO:0008234	O16288,O16289,P43508,P43509,O45466,P25807,P43507,P43510	PEPT_C1 DOMAIN-CONTAINING PROTEIN (PTHR12411:SF281)	cysteine protease(PC00081)
Minc3s00643g15540	GO:0006508,GO:0004222	O16796,Q18673,O44857	NEPRILYSIN-11 (PTHR11733:SF205)	metalloprotease(PC00153)
Minc3s02143g28529	GO:0006355,GO:0043565,GO:0003700	O16850	FORKHEAD BOX PROTEIN O (PTHR45767:SF2)	-
Minc3s03756g34708	GO:0006355,GO:0043565,GO:0003700	O16850	FORKHEAD BOX PROTEIN O (PTHR45767:SF2)	-
Minc3s00166g06546	GO:0006355,GO:0043401,GO:0005634,GO:0008270,GO:0003700,GO:0043565,GO:0003707	O16890,O17611	NUCLEAR HORMONE RECEPTOR FAMILY-RELATED (PTHR24083:SF88)	C4 zinc finger nuclear receptor(PC00169)
Minc3s00249g08550	GO:0006355,GO:0043401,GO:0005634,GO:0008270,GO:0003700,GO:0043565,GO:0003707	O16890,O17611	NUCLEAR HORMONE RECEPTOR FAMILY-RELATED (PTHR24083:SF88)	C4 zinc finger nuclear receptor(PC00169)
Minc3s00836g17930	GO:0055114,GO:0009055,GO:0016705,GO:0020037,GO:0005506	O17624,O17806,O17807,Q09653,Q27513,Q27514,Q27515,Q27516,Q27517,Q27518,Q27519,Q27520,Q9XUT8,Q1HB05,Q21262,Q27476,Q27477,Q27479,O17329	CYTOCHROME P450 FAMILY-RELATED (PTHR24282:SF7)	oxygenase(PC00177)
Minc3s00641g15504	GO:0006508,GO:0008270,GO:0004181	O17754	CARBOXYPEPTIDASE E (PTHR11532:SF62)	protease(PC00190)
Minc3s02499g30332	GO:0006508,GO:0008270,GO:0004181	O17754	CARBOXYPEPTIDASE E (PTHR11532:SF62)	protease(PC00190)

Minc3s02917g32057	GO:0006508,GO:0008270,GO:0004181	O17754	CARBOXYPEPTIDASE E (PTHR11532:SF62)	protease(PC00190)
Minc3s01348g23001	GO:0007186,GO:0016021,GO:0005887,GO:0004930,GO:0004993	O44986	G_PROTEIN_RECEP_F1_2 DOMAIN-CONTAINING PROTEIN (PTHR24247:SF249)	G-protein coupled receptor(PC00021)
Minc3s02701g31246	GO:0007186,GO:0016021,GO:0005887,GO:0004930,GO:0004993	O44986	G_PROTEIN_RECEP_F1_2 DOMAIN-CONTAINING PROTEIN (PTHR24247:SF249)	G-protein coupled receptor(PC00021)
Minc3s00704g16335	GO:0005509	O45313	EF-HAND DOMAIN-CONTAINING PROTEIN (PTHR23055:SF171)	-
Minc3s02094g28247	GO:0005509	O45313	EF-HAND DOMAIN-CONTAINING PROTEIN (PTHR23055:SF171)	-
Minc3s01372g23237	GO:0006457	O45418	12 KDA FK506-BINDING PROTEIN-RELATED (PTHR10516:SF443)	chaperone(PC00072)
Minc3s00048g02611	GO:0005515	O45451,P91252,P91253,P91254,Q09596,Q21743,Q23520,Q21945	GLUTATHIONE S-TRANSFERASE (PTHR11571:SF132)	transferase(PC00220)
Minc3s00365g11068	GO:0005515	O45451,P91252,P91253,P91254,Q09596,Q21743,Q23520,Q21945	GLUTATHIONE S-TRANSFERASE (PTHR11571:SF132)	transferase(PC00220)
Minc3s00103g04620	GO:0046983	O45489,Q7YWS9,Q86D08	BHLH DOMAIN-CONTAINING PROTEIN (PTHR19290:SF104)	basic helix-loop-helix transcription factor(PC00055)
Minc3s00096g04440	GO:0008289	P34382,P34383,Q9XUB7	FATTY-ACID AND RETINOL-BINDING PROTEIN 1 (PTHR31418:SF7)	
Minc3s00113g04971	GO:0008289	P34382,P34383,Q9XUB7	FATTY-ACID AND RETINOL-BINDING PROTEIN 1 (PTHR31418:SF7)	
Minc3s00696g16213	GO:0006468,GO:0035556,GO:0005524,GO:0005515,GO:0004672,GO:0004697,GO:0004674,GO:0004713,GO:0016772	P34722,P34885,P90980	PROTEIN KINASE C DELTA TYPE HOMOLOG-RELATED (PTHR24356:SF347)	non-receptor serine/threonine protein kinase(PC00167)
Minc3s01879g26926	GO:0016020,GO:0004104,GO:0004091	P38433	ACETYLCHOLINESTERASE 1 (PTHR43918:SF12)	esterase(PC00097)

Minc3s01629g25219	GO:0007218	P41855	FMRFAMIDE-RELATED PEPTIDES (PTHR20986:SF22)	-
Minc3s01119g20894	GO:0015696,GO:0072488,GO:0016020,GO:0008519	P54145,Q17663	AMMONIUM TRANSPORTER 1-RELATED (PTHR11730:SF6)	primary active transporter(PC00068)
Minc3s01133g21042	GO:0016020	P83386,P83387	MARVEL DOMAIN-CONTAINING PROTEIN (PTHR22776:SF49)	membrane traffic protein(PC00150)
Minc3s00018g01178	GO:0006518,GO:0055114,GO:0016020,GO:0005507,GO:0005515,GO:0004504	P91268	PEPTIDYL-GLYCINE ALPHA-AMIDATING MONOOXYGENASE (PTHR10680:SF14)	oxygenase(PC00177)
Minc3s00479g13034	GO:0005515	P91493	FI18813P1 (PTHR10024:SF369)	membrane trafficking regulatory protein(PC00151)
Minc3s03902g35102	GO:0005515,GO:0008270,GO:0046872	Q03601	RING-TYPE DOMAIN-CONTAINING PROTEIN (PTHR24104:SF38)	ubiquitin-protein ligase(PC00234)
Minc3s00289g09501	GO:0016747	Q09225	NOSE RESISTANT TO FLUOXETINE PROTEIN 6 (PTHR11161:SF65)	acyltransferase(PC00042)
Minc3s00582g14621	GO:0045454,GO:0006662,GO:0009055,GO:0015035	Q09433,Q9N357,Q9TXY8	THIOREDOXIN (PTHR10438:SF423)	oxidoreductase(PC00176)
Minc3s00195g07236	GO:0038032,GO:0006468,GO:0007165,GO:0005524,GO:0004703,GO:0004672,GO:0004674,GO:0016772	Q09537	G PROTEIN-COUPLED RECEPTOR KINASE 2 (PTHR24355:SF28)	non-receptor serine/threonine protein kinase(PC00167)
Minc3s01113g20838	GO:0038032,GO:0006468,GO:0007165,GO:0005524,GO:0004703,GO:0004672,GO:0004674,GO:0016772	Q09537	G PROTEIN-COUPLED RECEPTOR KINASE 2 (PTHR24355:SF28)	non-receptor serine/threonine protein kinase(PC00167)
Minc3s02470g30210	GO:0055114,GO:0016491,GO:0005506,GO:0010181	Q09590	NADPH--CYTOCHROME P450 REDUCTASE (PTHR19384:SF17)	oxidoreductase(PC00176)
Minc3s02684g31181	GO:0006835,GO:0016020,GO:0017153	Q10901,Q22682	EXCITATORY AMINO ACID TRANSPORTER (PTHR11958:SF103)	primary active transporter(PC00068)
Minc3s01398g23484	GO:0006468,GO:0005524,GO:0004672,GO:0004713,GO:0016772	Q11090	SERINE/THREONINE-PROTEIN KINASE C01C4.3-RELATED (PTHR24361:SF837)	-

Minc3s00886g18507	GO:0008152,GO:0016758	Q17399	UDP-GLUCURONOSYLTRANSFERASE (PTHR48043:SF143)	-
Minc3s02950g32178	GO:0006810,GO:0016020,GO:0005215	Q17758,Q21219	SOLUTE CARRIER FAMILY 15 MEMBER 2 (PTHR11654:SF595)	transporter(PC00227)
Minc3s01046g20159	GO:0006468,GO:0005515,GO:0005543,GO:0004672,GO:0005524,GO:0004674,GO:0004713,GO:0016772	Q17941,Q9XTG7	RAC SERINE/THREONINE-PROTEIN KINASE (PTHR24356:SF333)	non-receptor serine/threonine protein kinase(PC00167)
Minc3s03504g34034	GO:0006468,GO:0005515,GO:0005543,GO:0004672,GO:0005524,GO:0004674,GO:0004713,GO:0016772	Q17941,Q9XTG7	RAC SERINE/THREONINE-PROTEIN KINASE (PTHR24356:SF333)	non-receptor serine/threonine protein kinase(PC00167)
Minc3s07575g41278	GO:0005543,GO:0005515	Q17941,Q9XTG7	RAC SERINE/THREONINE-PROTEIN KINASE (PTHR24356:SF333)	non-receptor serine/threonine protein kinase(PC00167)
Minc3s06764g40290	GO:0007186,GO:0016021,GO:0004930,GO:0004983	Q18179	NEUROPEPTIDE F RECEPTOR (PTHR24235:SF12)	G-protein coupled receptor(PC00021)
Minc3s04791g37005	GO:0006355,GO:0043401,GO:0005634,GO:0003707,GO:0003700	Q18192	NUCLEAR HORMONE RECEPTOR FAMILY MEMBER NHR-31 (PTHR47519:SF1)	C4 zinc finger nuclear receptor(PC00169)
Minc3s00350g10768	GO:0005543,GO:0005515	Q18820	-	-
Minc3s00274g09178	GO:0006470,GO:0016311,GO:0016791,GO:0008138	Q19388	PROTEIN CBG14753 (PTHR45961:SF3)	-
Minc3s01242g22006	GO:0006470,GO:0016311,GO:0016791,GO:0008138	Q19388	PROTEIN CBG14753 (PTHR45961:SF3)	-
Minc3s01039g20092	GO:0007186,GO:0016021,GO:0004930	Q19399	G_PROTEIN_RECEP_F1_2 DOMAIN-CONTAINING PROTEIN (PTHR24230:SF154)	G-protein coupled receptor(PC00021)
Minc3s02316g29414	GO:0055114,GO:0005507,GO:0016491	Q19687	FI03373P-RELATED (PTHR11709:SF438)	oxidase(PC00175)
Minc3s01210g21755	GO:0007264,GO:0035556,GO:0005622,GO:0005085	Q19770	RAP GUANINE NUCLEOTIDE EXCHANGE FACTOR HOMOLOG (PTHR23113:SF252)	guanyl-nucleotide exchange factor(PC00113)
Minc3s00014g00871	GO:0005509	Q20203	CALSEQUESTRIN (PTHR10033:SF0)	calcium-binding protein(PC00060)

Minc3s00338g10495	GO:0005861	Q20334,Q9GYF1,Q9XUN9	TROPONIN I 1 (PTHR13738:SF6)	non-motor actin binding protein(PC00165)
Minc3s01175g21423	GO:0005515	Q21465	IG-LIKE DOMAIN-CONTAINING PROTEIN (PTHR45080:SF18)	-
Minc3s01999g27675	GO:0005515	Q21465	IG-LIKE DOMAIN-CONTAINING PROTEIN (PTHR45080:SF18)	-
Minc3s01266g22271	GO:0005515	Q21624	CORONIN (PTHR10856:SF0)	non-motor actin binding protein(PC00165)
Minc3s03081g32699	GO:0015671,GO:0019825,GO:0020037,GO:0005506	Q21978	-	-
Minc3s00337g10479	GO:0005515	Q22422,Q9XUB9	GON-2 EXTRAGENIC MODIFIER (PTHR10857:SF132)	-
Minc3s03546g34156	GO:0033961,GO:0003824	Q23068	EPOXIDE HYDROLASE 1 (PTHR21661:SF35)	hydrolase(PC00121)
Minc3s00725g16567	GO:0019509,GO:0005737,GO:0046872	Q23261	METHYLTHIORIBULOSE-1-PHOSPHATE DEHYDRATASE (PTHR10640:SF7)	dehydratase(PC00091)
Minc3s04314g36017	GO:0007186,GO:0016021,GO:0004930	Q23305	G_PROTEIN_RECEP_F1_2 DOMAIN-CONTAINING PROTEIN (PTHR24235:SF23)	G-protein coupled receptor(PC00021)
Minc3s00012g00808	GO:0006937,GO:0005861	Q27371,Q7Z072	TROPONIN T (PTHR11521:SF12)	actin binding motor protein(PC00040)
Minc3s00889g18533	GO:0006937,GO:0005861	Q27371,Q7Z072	TROPONIN T (PTHR11521:SF12)	actin binding motor protein(PC00040)
Minc3s01028g20001	GO:0006730,GO:0008270,GO:0004089	Q27504	CARBONIC ANHYDRASE (PTHR18952:SF141)	-
Minc3s05749g38766	GO:0006730,GO:0008270,GO:0004089	Q27504	CARBONIC ANHYDRASE (PTHR18952:SF141)	-
Minc3s01906g27097	GO:0044237,GO:0006012,GO:0050662,GO:0003978,GO:0003824	Q564Q1	UDP-GLUCOSE 4-EPIMERASE (PTHR43725:SF8)	epimerase/racemase(PC00096)
Minc3s02808g31643	GO:0007165,GO:0005515	Q564X8	RAW, ISOFORM A (PTHR36300:SF1)	-
Minc3s03929g35162	GO:0007186,GO:0016021,GO:0005887,GO:0004930,GO:0004993	Q7JP61	G_PROTEIN_RECEP_F1_2 DOMAIN-CONTAINING PROTEIN (PTHR24247:SF247)	G-protein coupled receptor(PC00021)

Minc3s04191g35703	GO:0007186,GO:0016021,GO:0005887,GO:0004930,GO:0004993	Q7JP61	G_PROTEIN_RECEP_F1_2 DOMAIN-CONTAINING PROTEIN (PTHR24247:SF247)	G-protein coupled receptor(PC00021)
Minc3s00128g05455	GO:0007186,GO:0016021,GO:0004930	Q7YXG8	G_PROTEIN_RECEP_F1_2 DOMAIN-CONTAINING PROTEIN (PTHR24238:SF41)	G-protein coupled receptor(PC00021)
Minc3s00374g11206	GO:0007186,GO:0016021,GO:0004930	Q86ME6	DOPAMINE RECEPTOR 1 (PTHR24247:SF238)	G-protein coupled receptor(PC00021)
Minc3s00227g08053	GO:0007165,GO:0008081,GO:0004114	Q8I0P7	3',5'-CYCLIC PHOSPHODIESTERASE PDE-3-RELATED (PTHR11347:SF179)	phosphodiesterase(PC00185)
Minc3s02259g29120	GO:0016021	Q93249	NEURAL PROLIFERATION DIFFERENTIATION AND CONTROL PROTEIN 1 (PTHR23352:SF2)	-
Minc3s02754g31454	GO:0006470,GO:0016311,GO:0016791,GO:0008138,GO:0004725	Q93592	PROTEIN CBG00285 (PTHR45961:SF9)	-
Minc3s03507g34048	GO:0006470,GO:0016311,GO:0016791,GO:0008138,GO:0004725	Q93592	PROTEIN CBG00285 (PTHR45961:SF9)	-
Minc3s08970g42806	GO:0006470,GO:0016311,GO:0016791,GO:0008138,GO:0004725	Q93592	PROTEIN CBG00285 (PTHR45961:SF9)	-
Minc3s02994g32349	GO:0008270	Q95PW6	-	-
Minc3s02012g27750	GO:0005515	Q95PZ3	KELCH DOMAIN-CONTAINING PROTEIN 10 (PTHR46428:SF1)	-
Minc3s00009g00631	GO:0006164,GO:0003937,GO:0004643,GO:0003824	Q95QQ4	BIFUNCTIONAL PURINE BIOSYNTHESIS PROTEIN ATIC (PTHR11692:SF0)	-
Minc3s00005g00328	GO:0055114,GO:0006518,GO:0016020,GO:0004497,GO:0005507,GO:0016715,GO:0004504,GO:0003824	Q95XM2	PEPTIDYLGLYCINE ALPHA-HYDROXYLATING MONOOXYGENASE (PTHR10680:SF35)	oxygenase(PC00177)

Minc3s00380g11338	GO:0055114,GO:0006518,GO:0016020,GO:0004497,GO:0005507,GO:0016715,GO:0004504,GO:0003824	Q95XM2	PEPTIDYLGLYCINE ALPHA-HYDROXYLATING MONOOXYGENASE (PTHR10680:SF35)	oxygenase(PC00177)
Minc3s00702g16302	GO:0035556,GO:0009190,GO:0016849	Q966F9	ADENYLATE CYCLASE (PTHR45627:SF16)	-
Minc3s01101g20732	GO:0030554	Q9GYL2	METAL TRANSPORTER CNNM-2 (PTHR12064:SF23)	-
Minc3s01251g22098	GO:0007186,GO:0016021,GO:0004930	Q9U320	G_PROTEIN_RECEP_F1_2 DOMAIN-CONTAINING PROTEIN (PTHR24230:SF120)	G-protein coupled receptor(PC00021)
Minc3s01764g26130	GO:0007218,GO:0030141	Q9XTY3	NEUROENDOCRINE PROTEIN 7B2 (PTHR12738:SF0)	chaperone(PC00072)
Minc3s01914g27164	GO:0007218,GO:0030141	Q9XTY3	NEUROENDOCRINE PROTEIN 7B2 (PTHR12738:SF0)	chaperone(PC00072)
Minc3s00536g13924	GO:0006470,GO:0016311,GO:0016791,GO:0008138,GO:0004725	Q9XVE7	IP21249P (PTHR45961:SF6)	-
Minc3s00740g16731	GO:0006470,GO:0016311,GO:0016791,GO:0008138,GO:0004725	Q9XVE7	IP21249P (PTHR45961:SF6)	-
Minc3s05834g38890	GO:0006470,GO:0016311,GO:0016791,GO:0008138,GO:0004725	Q9XVE7	IP21249P (PTHR45961:SF6)	-
Minc3s00242g08382	GO:0007186,GO:0016021,GO:0004930	Q9XVQ0	G_PROTEIN_RECEP_F1_2 DOMAIN-CONTAINING PROTEIN (PTHR24229:SF96)	G-protein coupled receptor(PC00021)
Minc3s03289g33423	GO:0007186,GO:0016021,GO:0004930	Q9XVQ0	G_PROTEIN_RECEP_F1_2 DOMAIN-CONTAINING PROTEIN (PTHR24229:SF96)	G-protein coupled receptor(PC00021)
Minc3s00281g09313	GO:0006810,GO:0016020,GO:0005215	Q9XW36	AQUAPORIN OR AQUAGLYCEROPORIN RELATED (PTHR43829:SF9)	transporter(PC00227)

Minc3s03560g34196	GO:0006629,GO:0035556,GO:0007165,GO:0008081,GO:0005509,GO:0004435,GO:0005515	Q9XWB7	PHOSPHOINOSITIDE PHOSPHOLIPASE C (PTHR10336:SF193)	phospholipase(PC00186)
Minc3s00684g16046	GO:0005515	Q9XWM1	-	-
Minc3s00990g19638	GO:0007186,GO:0016021,GO:0004930	U4PMM5	G_PROTEIN_RECEP_F1_2 DOMAIN-CONTAINING PROTEIN (PTHR46641:SF1)	transmembrane signal receptor(PC00197)

Supplementary Table 5 | Transcriptional regulation of known subventral glands (SvG) effector genes. According to the literature [31], 48 non-redundant *M. incognita* effectors have been identified in SvG (i.e., columns: effector-gene number, gene name and accession number on M. *incognita* genome). For this study, SvG effector genes were classified according to both their expression level and flanking histone modifications during eggs-to-J2s transition. Differential gene expression is shown as RNA-seq fold expression changes, Log2(Fold Change), calculated using DESeq2 on triplicates, with a p value < 0.05 as a threshold for overexpression. Effector genes were considered to be associated with a histone modification if at least 1 bp of the annotation overlapped with an identified histone modification. Three biological replicates of M. incognita eggs and J2s have been treated jointly to identify common histone modification enrichment using ChromstaR. [] indicates no histone modification has been identified. NS indicates no difference in gene expression between egg and J2 samples. NA indicates no predicted genes on *M. incognita* genome.

Gene numbers according to Da Rocha et al., 2021	Gene names according to literature	Log2 (Fold-change J2s/Eggs)	Gene	HPTM_eggs	HPTM_J2s
Effector genes associated with a similar expression level during eggs-to-J2s transition					
1	16D10	NS	NA	[]	[]
4	2G06B (CM1)	NS	NA	[]	[]
11	5G06 (msp26)	NS	Minc3s00664g15812	[]	[]
12	6D09B (CM1, CM2, 2G06B)	NS	NA	[]	[]
16	8H11 (msp10, 10A08)	NS	Minc3s01802g26418	[]	[]
19	CL321Contig1_1-EST	NS	Minc3s00011g00761	[]	[]
20	CL480Contig2_1-EST	NS	Minc3s00475g12970	[]	[]
34	Minc03314	NS	Minc3s00020g01281	[]	[]
36	Minc03328	NS	Minc3s00020g01299	[]	[]
38	Minc04584	NS	NA	[]	[]
41	Minc10418 (Minc08073)	NS	Minc3s01112g20831	[]	[]
44	Minc15401	NS	Minc3s06052g39242	[]	[]
45	Minc18033 (16E05, msp17)	NS	Minc3s03024g32468	[]	[]
28	Mi-PNF3 (Profilin3)	NS	Minc3s00239g08307	[]	[]
30	Mi-Xyl1	NS	Minc3s00084g04025	[]	[]
32	Minc00469	NS	Minc3s00013g00811	[]	[H3K27me3]
40	Minc08146	NS	Minc3s00234g08205	[]	[H3K27me3]
42	Minc12024	NS	NA	[]	[H3K27me3+H3K9me3+H4K20me1]

15	8E10B (msp40)	NS	Minc3s05895g38985	[]	[H3K4me3]
9	4D01 (msp3)	NS	Minc3s01472g24105	[]	[H3K9me3]
46	Minc18636 (MiEFF18)	NS	Minc3s01525g24488	[]	[H3K9me3]
22	HM1	NS	Minc3s00001g00021	[H3K27ac]	[H3K27ac]
39	Minc08073 (Minc10418)	NS	Minc3s00371g11166	[H3K27ac]	[H3K27ac]
5	30G11 (msp21)	NS	Minc3s00271g09100	[H3K4me3]	[H3K4me3]
17	CL2552Contig1_1-EST	NS	Minc3s01149g21190	[H3K4me3]	[H3K4me3]
31	Minc00344 (Minc04584)	NS	Minc3s00056g02915	[H4K20me1]	[H4K20me1]
Effector genes associated with a differential expression level during eggs-to-J2s transition					
7	31H06 (msp22)	3.57	Minc3s00376g11251	[]	[]
47	SXP-RAL2=Mi-SXP-1	4.08	Minc3s00381g11354	[]	[]
27	Mi-PG1	5.59	Minc3s00007g00481	[]	[]
2	2B02B (Mi-PEL2)	6.12	Minc3s00094g04359	[]	[]
26	Mi-PEL2	6.40	Minc3s00566g14364	[]	[]
35	Minc03325	7.55	Minc3s00020g01295	[]	[]
21	CL5Contig2_1-EST (Sec-2)	2.30	Minc3s00113g04971	[H3K4me3]	[H3K4me3]
33	Minc01696	4.48	Minc3s00036g02098	[H3K9me3]	[H3K9me3]
Effector genes associated with both a histone modification dynamic and a differential expression level during eggs-to-J2s transition					
25	Mi-GSTS1	2.14	Minc3s00365g11068	[]	[H3K4me3]
6	30H07 (msp20)	2.95	Minc3s05190g37766	[]	[H3K27ac+H3K27me3+H4K20me1]
13	8D05 (msp9)	3.20	Minc3s01244g22037	[]	[H3K9me3]
18	CL312Contig1_1-EST	3.90	Minc3s00070g03486	[H3K4me3+H4K20me1]	[H3K4me3]
3	2G02 (msp2)	3.96	Minc3s00855g18130	[]	[H4K20me1]
14	8E08B (Eng4)	5.04	Minc3s00139g05823	[]	[H3K9me3]
48	Mi-PEL1	6.09	Minc3s00441g12378	[]	[H3K27me3]
8	34C04 (Mi-PL1)	6.14	Minc3s01107g20785	[H4K20me1]	[H3K27me3]
29	Mi-VAP2	6.33	Minc3s01051g20218	[]	[H3K27ac+H3K27me3+H4K20me1]
23	Mi-CBP1 (42G06)	6.51	Minc3s00139g05824	[H3K27me3]	[H3K9me3]
43	Minc13292	6.99	Minc3s00083g03979	[H3K9me3]	[]
10	5A12B (ENG1, ENG3)	8.51	Minc3s03138g32920	[]	[H3K27ac+H3K27me3+H4K20me1]
24	Mi-ENG1 (1C11B)	8.53	Minc3s03136g32914	[]	[H3K27me3+H4K20me1]
37	Minc03866	8.67	Minc3s00066g03327	[H3K27me3+H3K9me3+H4K20me1]	[H3K9me3]

Supplementary Table 6 | Transcriptional regulation of known dorsal gland (DG) effector genes. According to the literature [31], 34 non-redundant *M. incognita* effectors have been identified in DG (i.e. columns: effector-gene number, gene name and accession number on *M. incognita* genome). For this study, DG effector genes were classified according to both their expression level and flanking histone modifications during eggs-to-J2s transition. Differential gene expression is shown as RNA-seq fold expression changes, Log2(Fold Change), calculated using DESeq2 on triplicates, with a p value < 0.05 as a threshold for overexpression. Effector genes were considered to be associated with a histone modification if at least 1 bp of the annotation overlapped with an identified histone modification. Three biological replicates of *M. incognita* eggs and J2s have been treated jointly to identify common histone modification enrichment using ChromstaR. [] indicates no histone modification has been identified. NS indicates no difference in gene expression between egg and J2 samples. NA indicates no predicted genes on *M. incognita* genome.

Gene numbers according to Da Rocha et al., 2021	Gene names according to literature	Log2(Fold-change J2s/Eggs)	Gene	HPTM_egg	HPTM_juvenile
Effector genes associated with a similar expression level during eggs-to-J2s transition					
1	10G02 (msp29)	NS	Minc3s00046g02513	[]	[]
2	12H03 (msp13)	NS	NA	[]	[]
3	13A12 (2E07, 11A01, msp14)	NS	Minc3s00111g04880	[]	[]
4	17H02 (msp18)	NS	NA	[]	[]
6	1C05B (msp36)	NS	Minc3s01204g21688	[]	[]
7	1D08B (msp37)	NS	NA	[]	[]
8	21E02 (14E06, msp19)	NS	NA	[]	[]
10	2G10 (msp27)	NS	NA	[]	[]
11	34D01 (msp23)	NS	NA	[]	[]
13	35F03 (msp30)	NS	NA	[]	[]
14	4D03 (msp28)	NS	Minc3s00043g02427	[]	[]
19	7E12 (14E06, msp7)	NS	NA	[]	[]
20	7H08 (msp8)	NS	NA	[]	[]
21	9H10 (msp11)	NS	Minc3s00709g16396	[]	[]
27	Minc11817 (Minc18861)	NS	NA	[]	[]
29	Minc12754	NS	Minc3s00905g18741	[]	[]
30	Minc17611	NS	NA	[]	[]
31	Minc17998	NS	Minc3s01352g23040	[]	[]
32	Minc18876	NS	Minc3s00086g04105	[]	[]
33	28B04	NS	NA	NA	NA
5	19F07 (35E04, msp32)	NS	Minc3s00381g11350	[H3K27me3]	[H3K27me3]
15	5C03B (msp39)	NS	Minc3s00989g19628	[H3K9me3]	[H3K4me3]
17	6G07 (msp5)	NS	NA	[]	[H3K27me3+H4K20me1]
18	7A01 (msp6)	NS	Minc3s00173g06739	[]	[H3K9me3]

22	HM12	NS	Minc3s01504g24329	[]	[H3K4me3]
24	Minc00108	NS	Minc3s00001g00012	[]	[H3K27ac+H4K20me1]
34	4F05B	NS	NA	[H3K4me3]	[H3K4me3]
Effector genes associated with a differential expression level during eggs-to-J2s transition					
28	Minc12639	3.18	Minc3s00340g10545	[]	[]
25	Minc01595	3.90	Minc3s01184g21493	[H3K27me3+H4K20me1]	[H3K27me3+H4K20me1]
Effector genes associated with both a histone modification dynamic and a differential expression level during eggs-to-J2s transition					
12	34F06 (msp24)	2.68	Minc3s00321g10151	[H3K27me3]	[H3K27me3+H3K9me3]
26	Minc02097 (35A02, msp25)	4.80	Minc3s00202g07465	[]	[H3K27me3]
9	25B10 (msp33)	4.99	Minc3s03649g34419	[]	[H3K9me3]
16	6F06 (msp4)	5.65	Minc3s02324g29465	[H3K9me3]	[]
23	Mi-14-3-3	-3.43	Minc3s00122g05244	[]	[H3K27me3]
